# Unveiling mitophagy-mediated molecular heterogeneity and development of a risk signature model for colorectal cancer by integrated scRNA-seq and bulk RNA-seq analysis

**DOI:** 10.1093/gastro/goad066

**Published:** 2023-10-24

**Authors:** Han Gao, Qi Zou, Linyun Ma, Keyu Cai, Yi Sun, Li Lu, Donglin Ren, Bang Hu

**Affiliations:** Department of General Surgery (Coloproctology), The Sixth Affiliated Hospital, Sun Yat-sen University, Guangzhou, Guangdong, P. R. China; Guangdong Provincial Key Laboratory of Colorectal and Pelvic Floor Diseases, The Sixth Affiliated Hospital, Sun Yat-sen University, Guangzhou, Guangdong, P. R. China; Biomedical Innovation Center, The Sixth Affiliated Hospital, Sun Yat-sen University, Guangzhou, Guangdong, P. R. China; Department of General Surgery (Coloproctology), The Sixth Affiliated Hospital, Sun Yat-sen University, Guangzhou, Guangdong, P. R. China; Guangdong Provincial Key Laboratory of Colorectal and Pelvic Floor Diseases, The Sixth Affiliated Hospital, Sun Yat-sen University, Guangzhou, Guangdong, P. R. China; Biomedical Innovation Center, The Sixth Affiliated Hospital, Sun Yat-sen University, Guangzhou, Guangdong, P. R. China; Department of Anesthesiology, Sun Yat-sen Memorial Hospital, Sun Yat-sen University, Guangzhou, Guangdong, P. R. China; Department of General Surgery (Coloproctology), The Sixth Affiliated Hospital, Sun Yat-sen University, Guangzhou, Guangdong, P. R. China; Guangdong Provincial Key Laboratory of Colorectal and Pelvic Floor Diseases, The Sixth Affiliated Hospital, Sun Yat-sen University, Guangzhou, Guangdong, P. R. China; Biomedical Innovation Center, The Sixth Affiliated Hospital, Sun Yat-sen University, Guangzhou, Guangdong, P. R. China; Department of Pathology, Kingmed Pathology Center, Guangzhou, Guangdong, P. R. China; Department of General Surgery (Coloproctology), The Sixth Affiliated Hospital, Sun Yat-sen University, Guangzhou, Guangdong, P. R. China; Guangdong Provincial Key Laboratory of Colorectal and Pelvic Floor Diseases, The Sixth Affiliated Hospital, Sun Yat-sen University, Guangzhou, Guangdong, P. R. China; Biomedical Innovation Center, The Sixth Affiliated Hospital, Sun Yat-sen University, Guangzhou, Guangdong, P. R. China; Department of General Surgery (Coloproctology), The Sixth Affiliated Hospital, Sun Yat-sen University, Guangzhou, Guangdong, P. R. China; Guangdong Provincial Key Laboratory of Colorectal and Pelvic Floor Diseases, The Sixth Affiliated Hospital, Sun Yat-sen University, Guangzhou, Guangdong, P. R. China; Biomedical Innovation Center, The Sixth Affiliated Hospital, Sun Yat-sen University, Guangzhou, Guangdong, P. R. China; Department of General Surgery (Coloproctology), The Sixth Affiliated Hospital, Sun Yat-sen University, Guangzhou, Guangdong, P. R. China; Guangdong Provincial Key Laboratory of Colorectal and Pelvic Floor Diseases, The Sixth Affiliated Hospital, Sun Yat-sen University, Guangzhou, Guangdong, P. R. China; Biomedical Innovation Center, The Sixth Affiliated Hospital, Sun Yat-sen University, Guangzhou, Guangdong, P. R. China

**Keywords:** colorectal cancer, scRNA-seq, mitophagy, risk signature, anticancer therapy

## Abstract

**Background:**

Accumulating researchers have recognized mitophagy as a key player in tumors, but few studies have investigated its role in the tumor microenvironment (TME). Advances in the technology of single-cell RNA sequencing (scRNA-seq) have allowed unveiling the concealed features of the TME at cellular resolution. This study aimed to elucidate the role of mitophagy within the TME of colorectal cancer (CRC) and to establish a mitophagy-mediated risk model.

**Methods:**

We assessed mitophagy-related pathway activities at both single-cell and tissue levels. Subsequently, an unsupervised clustering algorithm was employed to identify mitophagy-mediated subtypes. Furthermore, we developed a mitophagy-mediated risk signature (MMRS) using least absolute shrinkage and selection operator (LASSO) Cox analysis and constructed a MMRS model incorporating the risk score and clinical variables. Subsequently, we used quantitative reverse transcription polymerase chain reaction analysis to verify the expression of the screened genes.

**Results:**

We retrieved and annotated a total of 14,719 cells from eight samples in the scRNA-seq GSE132465 data set. The activities of mitophagy-related pathways were uniformly upregulated in cancer cells. Integrating with bulk RNA-seq data, we identified two mitophagy-mediated clusters (C1 and C2) with distinct characteristics and prognoses. C2 was identified as a mitophagy-high cluster. Then, we developed a five-gene MMRS via LASSO Cox analysis in The Cancer Genome Atlas (TCGA) cohort. We utilized the GSE39582 cohort to validate the efficacy of our model. The expression of *CX3CL1* and *INHBB* was upregulated in CRC tissues.

**Conclusions:**

The present study identified two mitophagy-mediated CRC subtypes with distinct features. Our MMRS may provide potential therapeutic strategies for CRC. The findings of our work offer novel insights into the involvement of mitophagy in CRC.

## Introduction

Among all the malignancies, colorectal cancer (CRC) ranks third in incidence and is the second most frequent cause of deaths related to tumor, resulting in ∼2 million new diagnoses and 930,000 deaths per year [1, 2]. China had the highest incidence of new cases of CRC and the largest number of CRC-associated deaths in 2020 [3]. Moreover, CRC is highly heterogeneous, with varied clinicopathological and molecular characteristics, leading to different tumor progression statuses and varying treatment outcomes [4].

Mitophagy is a biological mechanism characterized by the selective removal of dysfunctional or senescent mitochondria via autophagy [5]. It is a key regulatory mechanism for removing damaged mitochondria and maintaining cellular balance [6]. Moreover, mitophagy serves a critical function in modulating metabolic reprogramming in cancer, maintaining cell stemness and fostering resistance to chemotherapy, thereby enhancing the adaptation of cancer cells to the tumor microenvironment (TME) [7]. Mitophagy is increasingly recognized as a key factor influencing the adaptability of cancer cells and their interaction with the TME. It facilitates metabolic reconfiguration in cancer stem cells (CSCs), thereby promoting their plasticity and adjusting to the TME better. For instance, mitophagy has been found to positively regulate the hepatic CSCs by inhibiting p53 activity [8]. A report by Valencia *et al*. [9] has shown that, in a prostate cancer model, the levels of p62 (a protein associated with mitophagy) are decreased in cancer-associated fibroblasts via deregulation of the mTORC1/c-Myc pathway. Acting as a tumor suppressor, p62/SQSTM1 promotes the removal of ubiquitinated dysfunctional mitochondria, particularly in inflammatory macrophages present within the TME [10]. The advent of single-cell RNA sequencing (scRNA-seq) technology has granted us the ability to dissect the intricate TME at the cellular level. The function of mitophagy in the TME of CRC warrants substantial clarification.

Mitophagy serves a dual role in cellular function. On the one hand, optimal mitophagy maintains cellular homeostasis and normal metabolism, and promotes cell survival to enhance adaptability to the invasive environment [11]. On the other hand, an overabundance of mitophagy can lead to disruption of cellular energy requirements and induction of cell death [12]. Targeting the mitophagy pathway could potentially tip the balance between cancer development and cell death. Consequently, either inhibiting or promoting mitophagy emerges as a strategy of considerable interest in the realm of cancer treatment. Drugs targeting mitochondria have been found to trigger cell death in a mitophagy-dependent manner, specifically through the AMPK-mTOR-ULK1 pathway, in colon cancer cells expressing both K-Ras wild-type and hyperactive mutant versions [13]. Given the crucial role that mitophagy plays in cancer, detecting specific biomarkers associated with mitophagy could be beneficial for targeting tumors. To illuminate the involvement in tumor progression and find promising therapeutic targets for CRC, in-depth research is imminently required.

In this work, we endeavored to explore the mitophagy-mediated tumor heterogeneity in CRC by integrating scRNA-seq and bulk RNA-seq data. The ultimate objective of our analysis was to gain further insight into the novel biomarkers for CRC and to provide references for targeted drug therapy. To achieve this, we constructed a novel mitophagy-mediated risk signature (MMRS) model for CRC patients and evaluated the influence of our MMRS on the therapeutic efficacy.

## Methods

### Data curation

**Figure 1.**
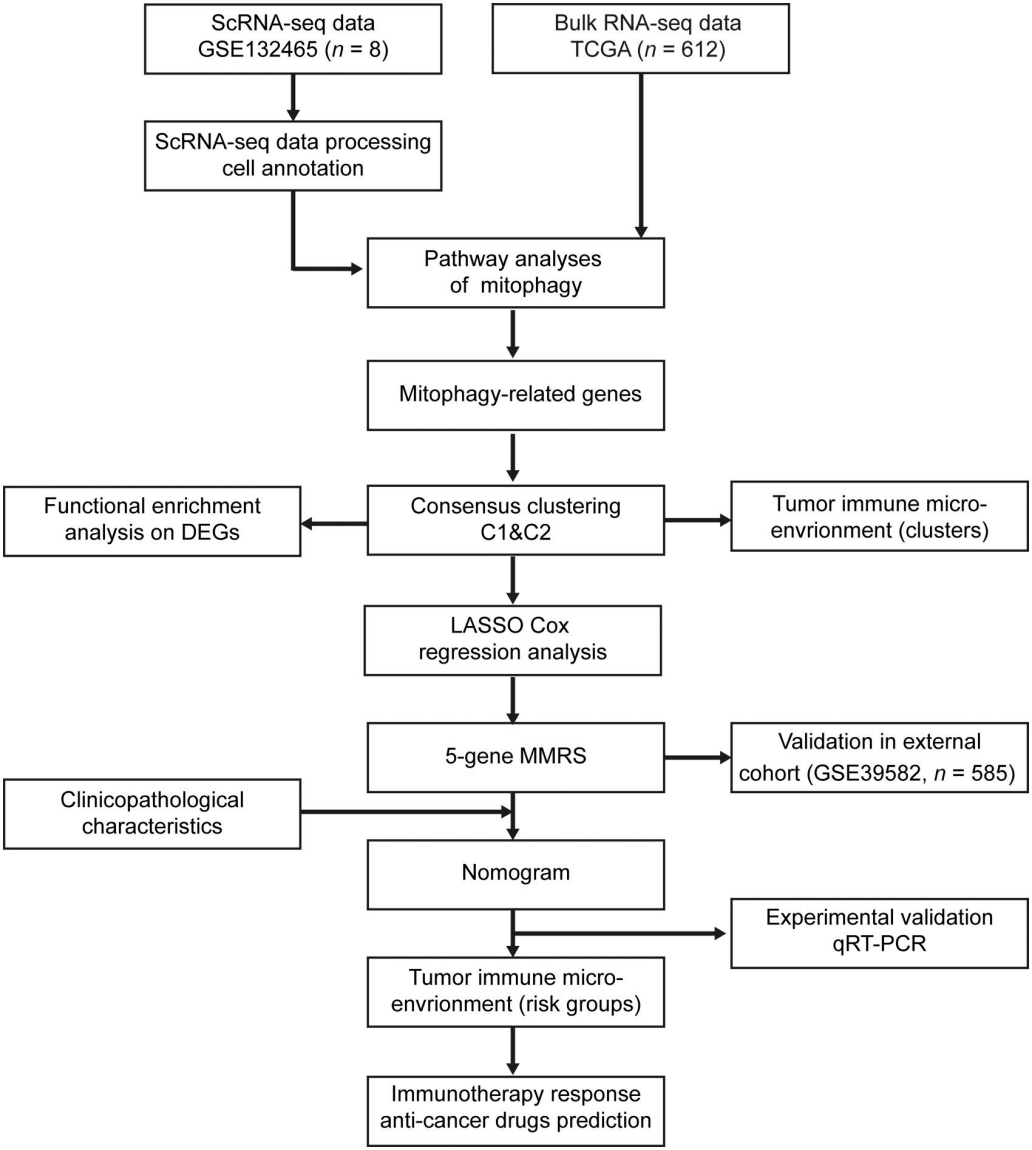
Workflow of this study. scRNA-seq = single-cell RNA sequencing, DEGs = differentially expressed genes, MMRS = mitophagy-mediated risk signature, qRT–PCR = quantitative reverse transcription–polymerase chain reaction, TCGA = The Cancer Genome Atlas.

We retrieved the scRNA-seq profile (GSE132465) from the Gene Expression Omnibus (GEO) database to depict the landscape of the immune microenvironment in CRC and to shed light on the integral role that mitophagy played within the TME. The bulk RNA-seq data (GSE39582 cohort, *n *=* *585; TCGA-COADREAD cohort, *n *=* *612) of CRC tumor samples, along with the relevant clinicopathological data, were acquired from the GEO and The Cancer Genome Atlas (TCGA) databases. The TCGA-COADREAD data set was employed as a training cohort, with the goal of identifying individual clusters and developing the MMRS model. Subsequently, we utilized the GSE39582 data set as a validation cohort to assess the performance and validity of our risk model. In addition, an annotated somatic mutation profile was retrieved from the TCGA repository. The workflow is illustrated in Figure 1.

### Data processing of scRNA-seq

We utilized the Seurat package (version 4.3.0) to transform the scRNA-seq count matrix data into Seurat objects [14]. To ensure data quality, cells with a high proportion of mitochondria genes (>3%) or low transcript count (<200 transcripts/cell) were filtered out. Subsequently, we normalized the scRNA-seq profile and identified the top 2,000 variable features via the FindVariableFeatures function. To effectively reduce the dimensionality and visualize the data, we utilized the RunPCA and RunTSNE functions to perform principal component analysis (PCA) and t-distributed stochastic neighbor embedding (t-SNE) analysis. Then, we used the FindAllMarkers function to identify the marker genes of each cluster and annotated each cell type based on the established studies. The CopyKAT function was employed to analyse copy number variations and identify aneuploid cells within the tumor. The cell clusters and cell types were visualized using the DimPlot function and the expression heat map for specific cells and features was generated using the DoHeatmap function.

### Activity evaluation of mitophagy-related pathways

We accessed the MSigDB database to obtain the pathways associated with mitophagy. The pathway activity was analysed using the escape package (version 1.10.0) and gene set variation analysis (GSVA) package (version 1.46.0) for scRNA-seq and bulk RNA-seq data, respectively [15]. By leveraging these analyses, we aimed to comprehensively examine the involvement of mitophagy-associated pathways in our study samples and gain insights into their potential functional roles in the context of our investigation.

### Unsupervised clustering

We first acquired the mitophagy-related genes and conducted a univariate Cox analysis to identify those with prognostic significance. Subsequently, these genes were utilized for unsupervised clustering analysis using the ConsensusClusterPlus package (version 1.62.0) [16]. This approach allowed us to classify samples into distinct groups based on their gene expression profiles, revealing underlying patterns and potential subtypes within the data set. A reliable clustering result was ensured by repeating the clustering procedure 1,000 times. The optimal number of clusters was identified using the cumulative distribution function (CDF) and CDF delta area. PCA plots for clusters were visualized using the ggplot2 package (version 3.4.2) [17]. We used Kaplan–Meier curves to verify the prognostic significance of the clustering results via the survminer package (version 0.4.9). We employed the limma package (version 3.56.2) to determine differentially expressed genes (DEGs) among the clusters according to an adjusted *P*-value of <0.05 and |logFC| of >1.

### Functional enrichment analysis

To gain further insights into the potential signaling pathways and biological processes related to DEGs, we performed enrichment analysis using the clusterProfiler package (version 4.6.2) [18]. This analysis involved the Gene Ontology (GO) and Kyoto Encyclopedia of Genes and Genomes (KEGG) databases. By exploring the enriched pathways and biological functions, we sought to unravel the underlying mechanisms associated with the DEGs. Moreover, to distinguish the biological profiles of the mitophagy-mediated clusters, we utilized the GSVA package (version 1.46.0) to conduct GSVA, which enabled us to assess the distinct biological characteristics of each mitophagy-mediated cluster.

### Mutational characteristics

We investigated the tumor mutation burden utilizing the maftools package (version 2.14.0) and created a waterfall plot to visualize the mutational characteristics across the different clusters [19].

### Depiction of the immune landscape

We carried out single sample gene set enrichment analysis (ssGSEA) to explore the TME and to depict the enrichment fraction of 23 immune cell types among clusters using the ggplot2 package (version 3.4.2). To compare the differences in immune cell infiltration among clusters, we performed the Estimation of STromal and Immune cells in MAlignant Tumor tissues using Expression data (ESTIMATE) algorithm to estimate the immune, stromal, and ESTIMATE scores. Moreover, to explore putative immunotherapy targets for CRC, we analysed the differences in the expression of immune checkpoint inhibitor (ICI) and human leukocyte antigen (HLA) genes among clusters.

### Development of the MMRS with prognostic value

To identify the prognostic genes, we applied univariate Cox analyses on the DEGs among the clusters via the glmnet package (version 4.1.4). Then, coefficients of each gene with predictive values were derived via LASSO Cox analysis [20]. The risk scores were calculated according to the formula as presented below:


$$\mathrm{risk}\;\mathrm{score}=\sum_{i=1}^{n}\left(\mathrm{coefi}*\mathrm{geneexpi}\right)$$

We classified CRC samples into the high-risk group (HRG) or the low-risk group (LRG) based on their median risk score. Subsequently, we plotted Kaplan–Meier curves to visualize the survival disparity between the HRG and the LRG. Additionally, we plotted a receiver-operating characteristic (ROC) curve and validated the effectiveness of our MMRS in predicting outcomes for CRC patients.

To determine whether the clinicopathological features or risk score were independently prognosis-associated values for CRC, we carried out univariate and multivariate Cox analyses. We utilized a validation cohort (GSE39582) to validate the capability and stability of the MMRS. Moreover, we utilized the pheatmap package (version 1.0.12) to illustrate the distribution of gene expression and clinicopathological characteristics between the HRG and the LRG.

### Nomogram

Integrating independent clinical variables (age, sex) and risk scores, we constructed a nomogram to quantify the prognostic variables and predict the overall survival (OS) probability of individuals. The predictive value of our nomogram was evaluated by plotting a calibration curve.

### Quantitative reverse transcription–polymerase chain reaction

A total of 15 pairs of CRC tumor and adjacent normal tissue samples were obtained from the Sixth Affiliated Hospital of Sun Yat-sen University for detecting the expression levels of *CX3CL1*, *CYP26A1*, *PNLDC1*, *INHBB*, and *PLIN1* by using quantitative reverse transcription–polymerase chain reaction (qRT–PCR). The qRT–PCR analysis was performed using the EZBioscences^®^ PCR system (EZBioscience, Roseville, CA, USA). The reaction started with an initial step of 95°C for 30 s, followed by 40 cycles of denaturation at 95°C for 10 s, annealing at 60°C for 30 s, and extension at 60°C for 30 s. The expression levels of each gene were determined using the 2^(−ΔΔCt) method, with GAPDH as the reference gene. Approval for the collection of patient samples was obtained from the Ethics Committee of the Sixth Affiliated Hospital of Sun Yat-sen University (granted number: 2022ZSLYEC-143). This study adheres to the principles outlined in the Declaration of Helsinki. The primer sequences for each gene are presented in Supplementary Table 1.

### GSEA

We employed the clusterProfiler package (version 4.6.2) to carry out GSEA and examine potential pathways enriched in the HRG. All pathways were ranked according to the normalized enrichment score; the eight most significant pathways were chosen for visualization (pathways were deemed enriched with a *P*-value of <0.05).

### Immunotherapy response and anticancer drugs prediction

To predict the response to immunotherapy, we extracted the immunophenotype of CRC samples from The Cancer Immunome Atlas to calculate the immunophenoscore. The immunophenoscore is positively correlated with the degree of immunogenicity. We estimated the ICI response between the HRG and the LRG by utilizing the tumor immune dysfunction and exclusion (TIDE) algorithm [21, 22]. We also used the oncoPredict package (version 0.2) to estimate the anticancer efficacy by evaluating the sensitivity to drugs in CRC patients from the Genomics of Drug Sensitivity in Cancer database [23].

## Results

### Single-cell analysis reveals tumor heterogeneity at the cellular level

**Figure 2.**
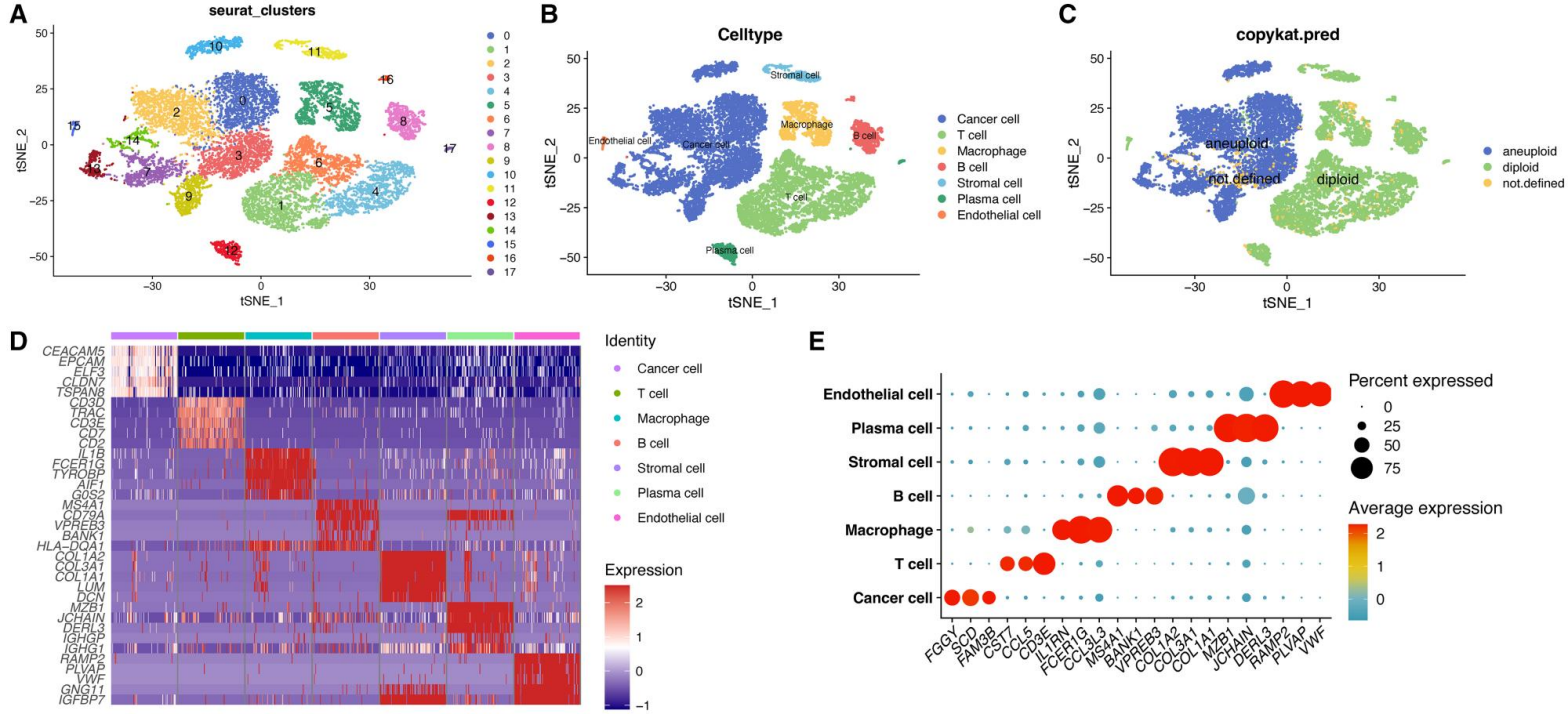
Processing of scRNA-seq data. (A) Cells were classified into 18 clusters. (B) Cell annotation. (C) Distribution of diploid cells and aneuploid cells. (D) Heat map displaying marker genes for each cell type. (E) Bubble plot demonstrating three marker genes for each cell type identified in this profile.

To characterize significant aspects of the intra-tumoral heterogeneity of CRC, we analysed the scRNA-seq data set GSE132465 to depict the landscape of the TME. Following quality control and normalization, 14,719 cells from eight tumor samples were filtered for further analyses (Supplementary Figure 1A and B). Two thousand genes were recognized as highly variable genes using the FindVariableFeatures function (Supplementary Figure 1C). PCA was applied to reduce dimensionality (Supplementary Figure 1D). Subsequently, we conducted FindNeighbors and FindClusters functions to classify all cells into 18 clusters, which were then annotated according to established studies (Figure 2A and B, and Supplementary Figure 1E). The result of the CopyKAT algorithm is presented in Figure 2C. The heat map and bubble plot serve to visualize the marker genes for each cell type (Figure 2D and E).

### Evaluation of mitophagy-related pathway activity

**Figure 3.**
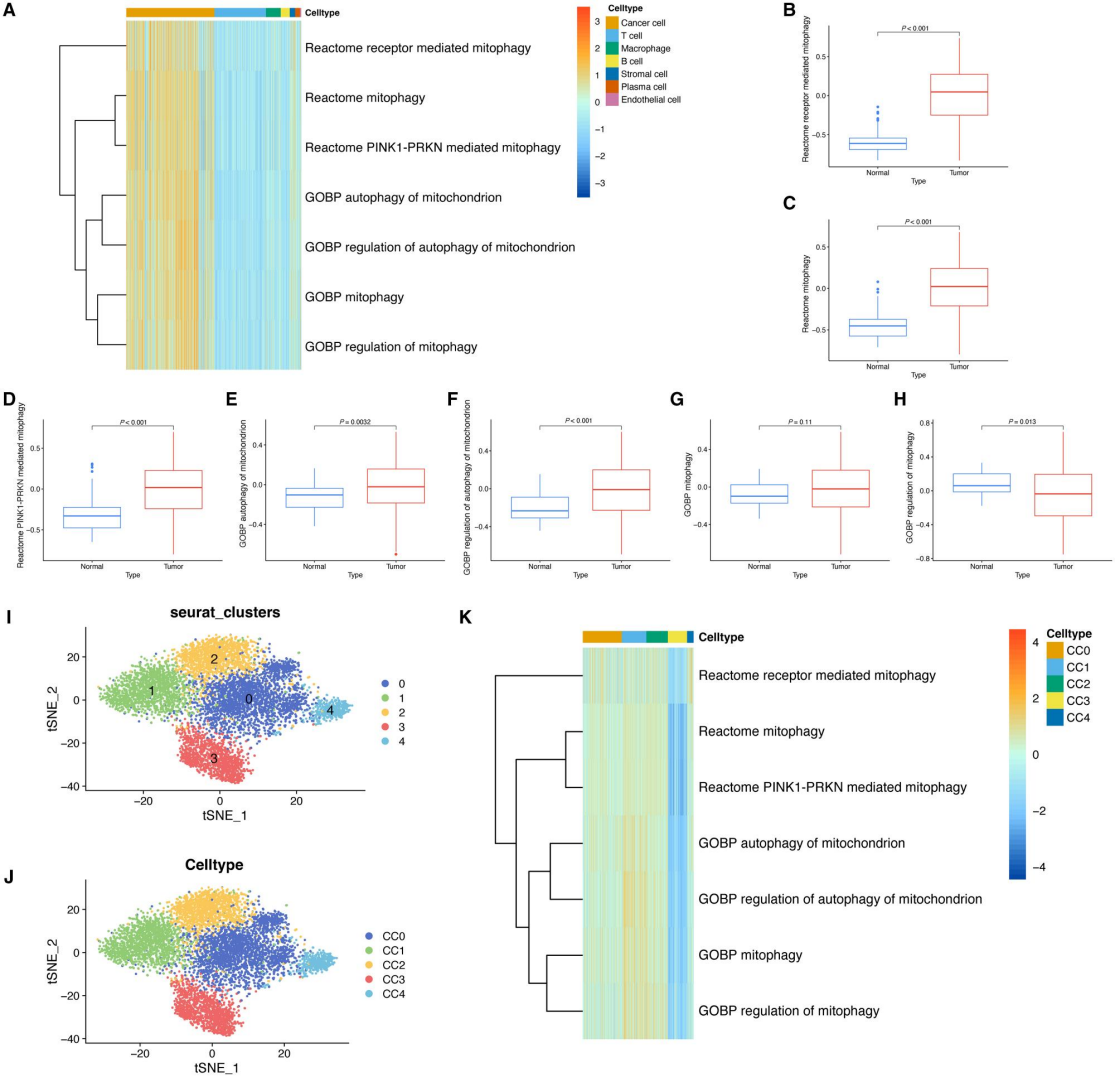
Evaluation of mitophagy-related pathways. (A) Heat map demonstrating that the activity of mitophagy-related pathways was upregulated in cancer cells. (B)–(H) Five out of seven mitophagy-related pathways were activated in the tumor. (I) Re-clustering of the cancer cell population. (H) Annotation of cancer cell subpopulations. (K) Heat map illustrating the diverse activity of mitophagy-related pathways in various cancer cell subpopulations. GOBP = Gene Ontology Biological Process.

We analysed the activity of seven mitophagy-related pathways by ssGSEA using scRNA-seq data. The heat map illustrated that the cancer cells exhibit a higher enrichment of mitophagy-related pathways (Figure 3A). In addition, we corroborated these findings through bulk RNA-seq data analysis, where five out of seven mitophagy-related pathways demonstrated higher activity levels in tumor tissues (Figure 3B–H). Consequently, these results demonstrated that mitophagy might be a key regulator for tumorigenesis and progression, and therefore warrant further investigation.

To further examine the role that mitophagy plays in the TME, we extracted the cancer cell population and re-clustered them based on the mitophagy-related genes, followed by annotation (Figure 3I and J). The subsequent pathway analysis of the cancer cell population revealed that the CC1 subpopulation displayed increased activity in pathways associated with mitophagy, while the CC3 subpopulation showed a downregulation of activity in these same pathways (Figure 3K).

### Classification of mitophagy-related clusters

**Figure 4.**
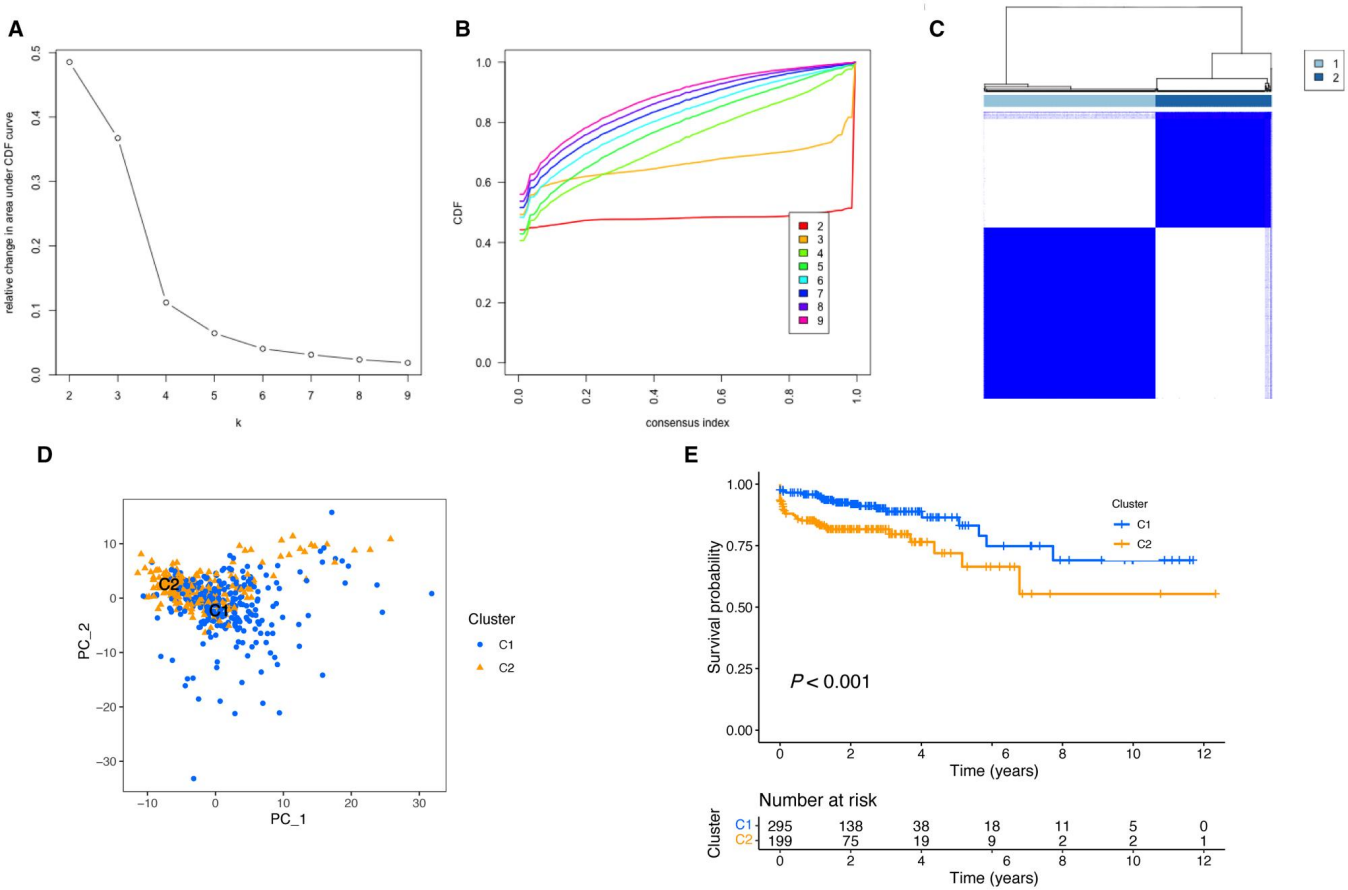
Uncovering of two mitophagy-related clusters for CRC. (A) Delta area. (B) Consensus clustering CDF. (C) Consensus matrix with *k* = 2. (D) PCA illustrating that the clusters were well distinguished. (E) Kaplan–Meier curve displaying the survival difference between the two clusters. CRC = colorectal cancer, CDF = cumulative distribution function, PCA = principal component analysis.

To unravel the molecular heterogeneity of CRC, we conducted consensus clustering analysis to classify samples of the training cohort (TCGA-COADREAD) into two distinct clusters according to the expression of 192 mitophagy-related genes (Supplementary Box), including cluster C1 (*n *=* *295) and cluster C2 (*n *=* *199, Figure 4A–C). PCA displayed that these clusters might be clearly discriminated based on the mitophagy-related genes (Figure 4D). Furthermore, patients in C2 demonstrated a poorer prognosis in comparison with those in C1 (Figure 4E).

### Functional enrichment analysis

**Figure 5.**
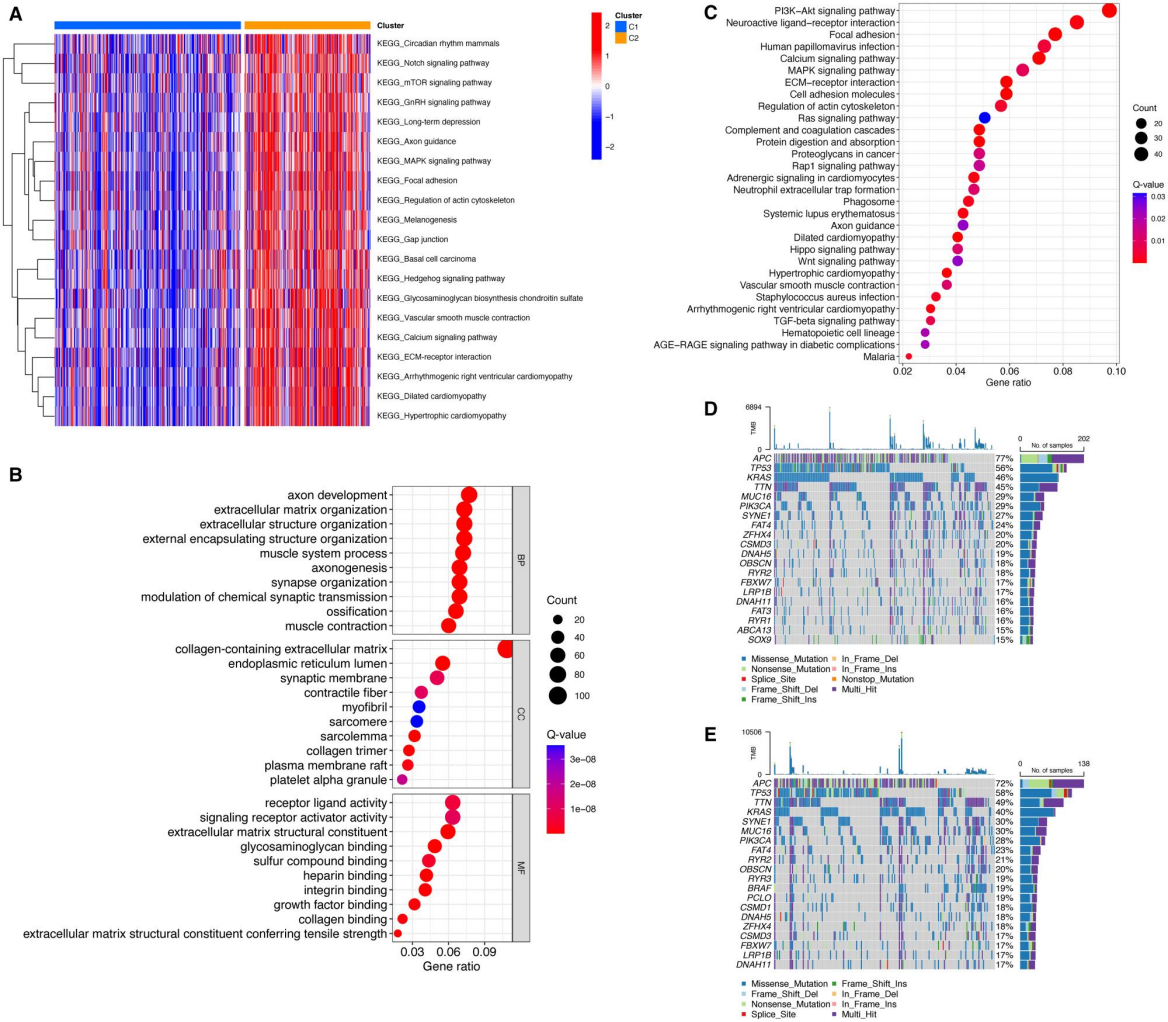
Functional and mutational analysis between C1 and C2. (A) Heat map showing the difference in GSVA between C1 and C2. (B) and (C) GO and KEGG analyses. (D) and (E) Waterfall plot illustrating the mutation landscape of C1 (mutations found in 252 out of 263 samples, accounting for 95.82%) and C2 (mutations observed in 186 out of 192 samples, accounting for 96.88%). GSVA = Gene Set Variation Analysis, GO = Gene Ontology, KEGG = Kyoto Encyclopedia of Genes and Genomes.

The GSVA substantiates the difference in enriched pathways between C1 and C2 (Figure 5A), providing further support for our findings. A total of 1,416 DEGs were identified between the two clusters and subsequently subjected to GO and KEGG analyses (Figure 5B and C). The differences in genetic mutations between the two clusters are displayed in the waterfall plot (Figure 5D and E).

### Immune landscape of C1 and C2

**Figure 6.**
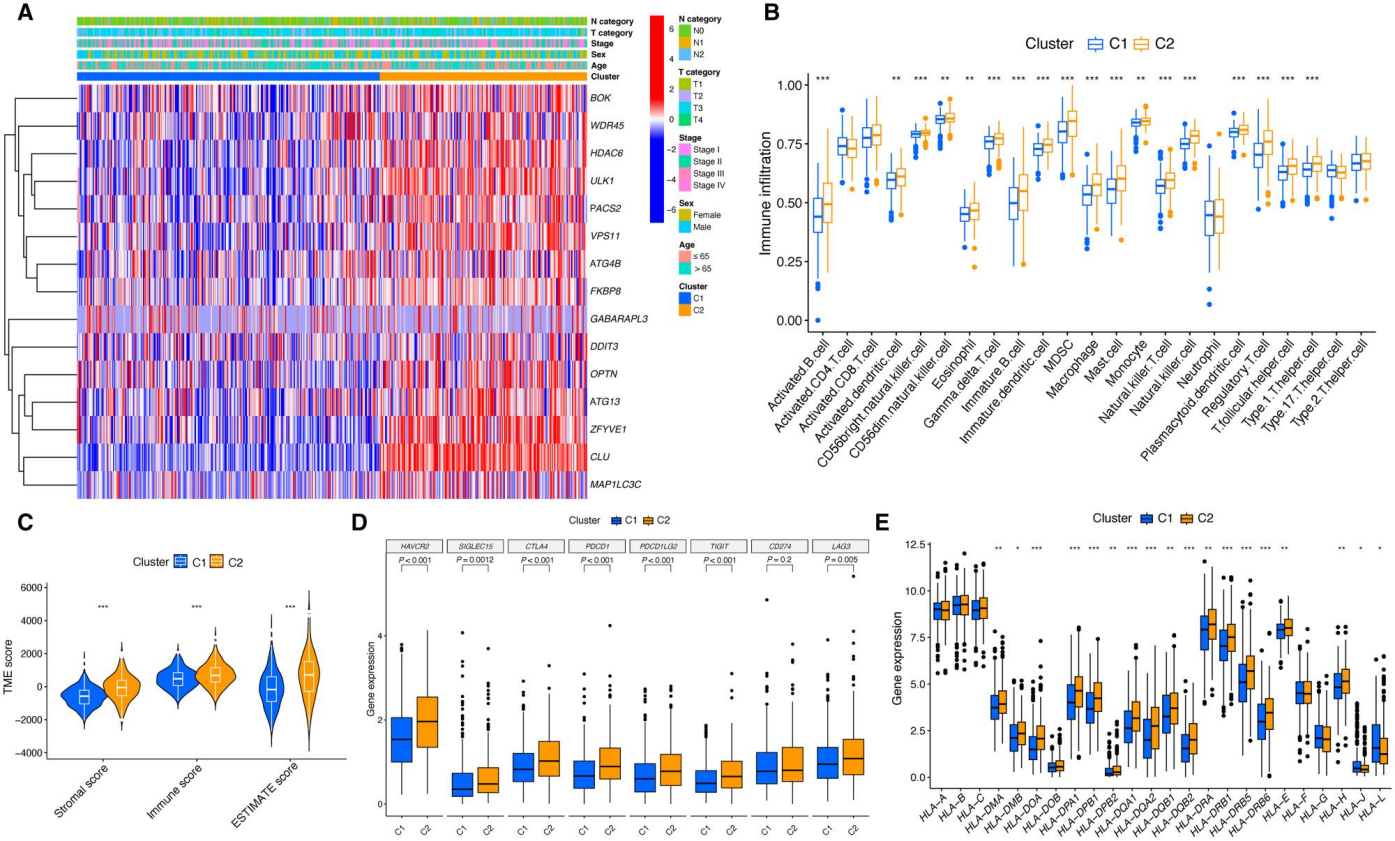
Immune infiltration analysis between C1 and C2. (A) Distribution of clinicopathological features and expression of prognostic mitophagy-related genes. (B) Differences in infiltration of 23 immune cells. (C) ESTIMATE analysis. (D) Difference in major ICI-related genes between clusters. (E) Difference in major HLA-related genes between clusters. TME = tumor microenvironment, ICI = immune checkpoint inhibitor, HLA = human leukocyte antigen, ESTIMATE = Estimation of STromal and Immune cells in MAlignant Tumor tissues using Expression data. *P < 0.05; **P < 0.01; ***P < 0.001.

We used a heat map to display the distribution of clinical variables and mitophagy-related gene expression with the prognostic value between the two clusters (Figure 6A). Results of ssGSEA illustrated that most immune cells were highly infiltrated in C2 (Figure 6B). To validate the robustness of the clustering results, we utilized the ESTIMATE algorithm to further unravel the TME according to the gene expression level of CRC samples. The results indicated that C2 exhibited consistently higher ESTIMATE, immune, and stromal scores compared with C1, indicating potential differences in the immune microenvironments between the two clusters (Figure 6C). Subsequently, we examined the expression of HLA- and ICI-related genes between the two clusters. HLA- and ICI-related genes are crucial for immunoreactivity and have varying clinical implications in the context of immunotherapy. The two clusters exhibited prominent differences in expression patterns of ICI- and HLA-related genes (Figure 6D and E).

### Construction of the MMRS model

**Figure 7.**
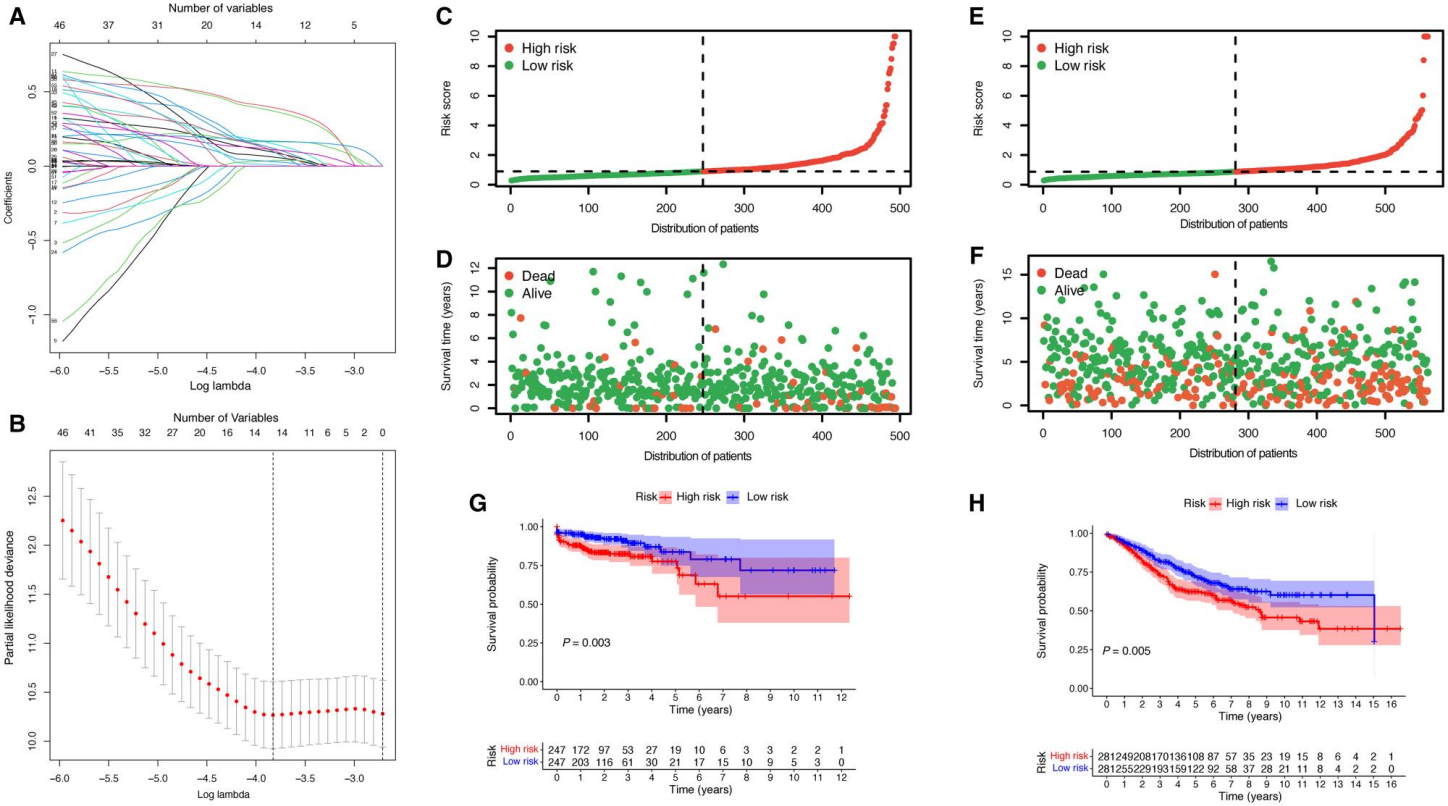
Development and validation of the MMRS. (A) and (B) Prognostic genes were obtained from LASSO Cox regression analysis. Distribution of risk scores and survival statuses in the (C) and (D) TCGA and (E) and (F) GSE39582 cohorts. Kaplan–Meier survival curve illustrating the survival difference in the (G) TCGA and (H) GSE39582 cohorts. MMRS = mitophagy-mediated risk signature, LASSO = least absolute shrinkage and selection operator, TCGA = The Cancer Genome Atlas.

To enhance the application of the subtypes in the precision therapy of CRC, we established a specific MMRS model according to DEGs between the two mitophagy-related clusters. Five genes were identified as independent prognostic genes via LASSO Cox regression analysis, namely *CX3CL1*, *CYP26A1*, *PNLDC1*, *INHBB*, and *PLIN1* (Figure 7A and B). Subsequently, we calculated the risk score for each sample utilizing the coefficients generated from the LASSO Cox analysis (Supplementary Table 2). Figure 7C–F illustrates the distribution of risk scores and survival status for both training and validation cohorts. The result of survival exhibited that the HRG suffered a poorer prognosis (Figure 7G). Consistently, we employed the external validation cohort (GSE39582) to corroborate the prognostic significance of our MMRS model (Figure 7H).

### Nomogram

**Figure 8.**
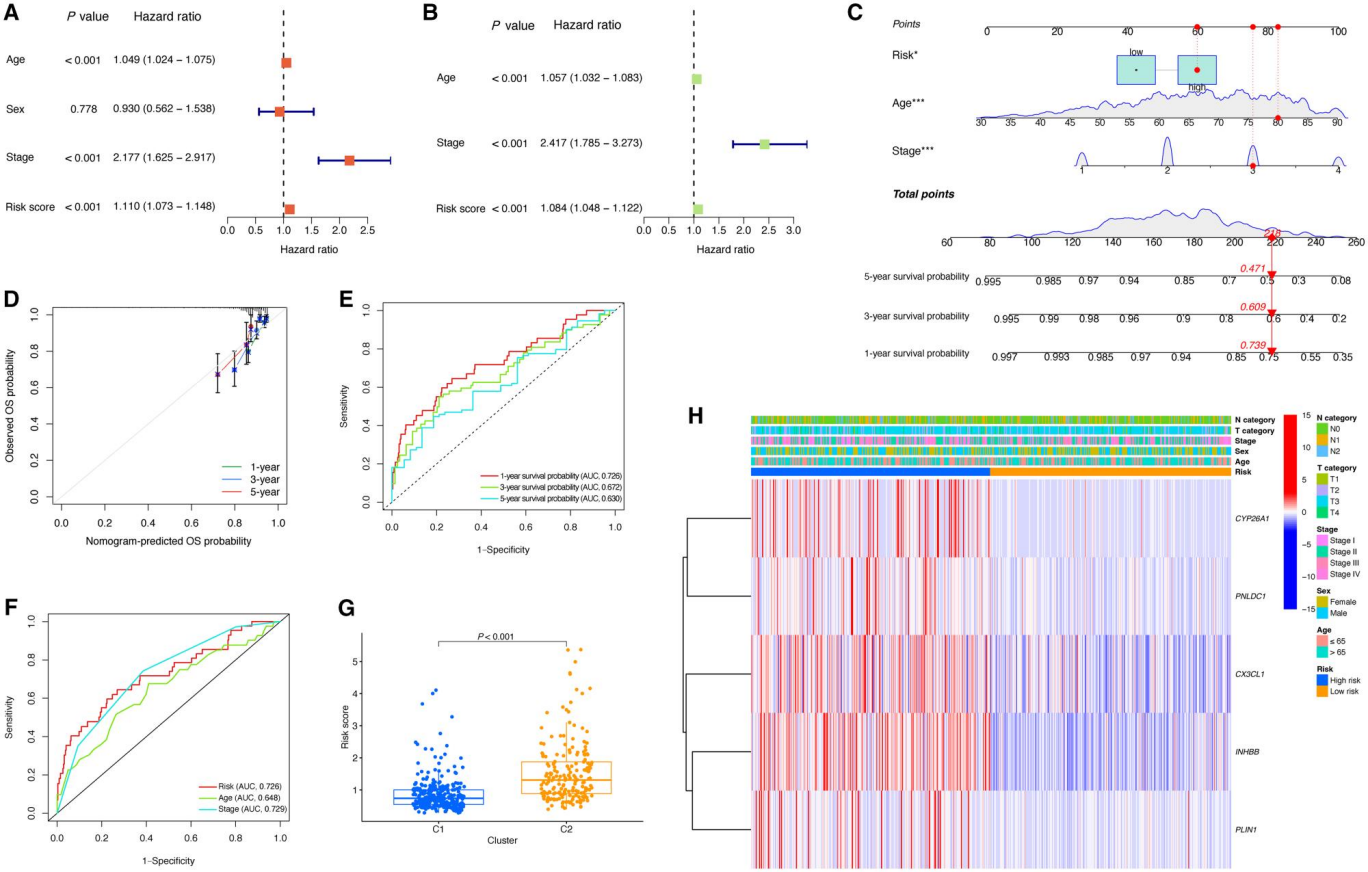
Nomogram. (A) and (B) Cox analyses for clinical variables and risk score. (C) Nomogram that was constructed. (D) Calibration curve showing a reliable performance of the model. (E) and (F) ROC curves for predicting the OS probability. (G) C2 exhibited a higher risk score compared with C1. (H) Heat map demonstrating the distribution of clinicopathological characteristics and expression of prognostic genes between the two groups. ROC = receiver-operating characteristic, OS = overall survival.

Based on the Cox analyses, the risk score was determined to be independently associated with the prognosis for CRC patients (Figure 8A and B). Next, we established the nomogram incorporating clinical variables and risk score, which could serve to visualize the survival probability for CRC patients (Figure 8C). The calibration curve demonstrated an excellent performance of our nomogram (Figure 8D). The area under the curve of the ROC for 1-, 3-, and 5-year survival was 0.726, 0.672, and 0.630, showing promising reliability for the model (Figure 8E). The ROC curve for clinical characteristics is shown in Figure 8F. Moreover, we observed that C2 exhibited a higher risk score compared with C1 (Figure 8G). Furthermore, the clinical variables and gene expression in HRG and LRG are displayed in a heat map (Figure 8H).

### qRT–PCR analysis

**Figure 9.**
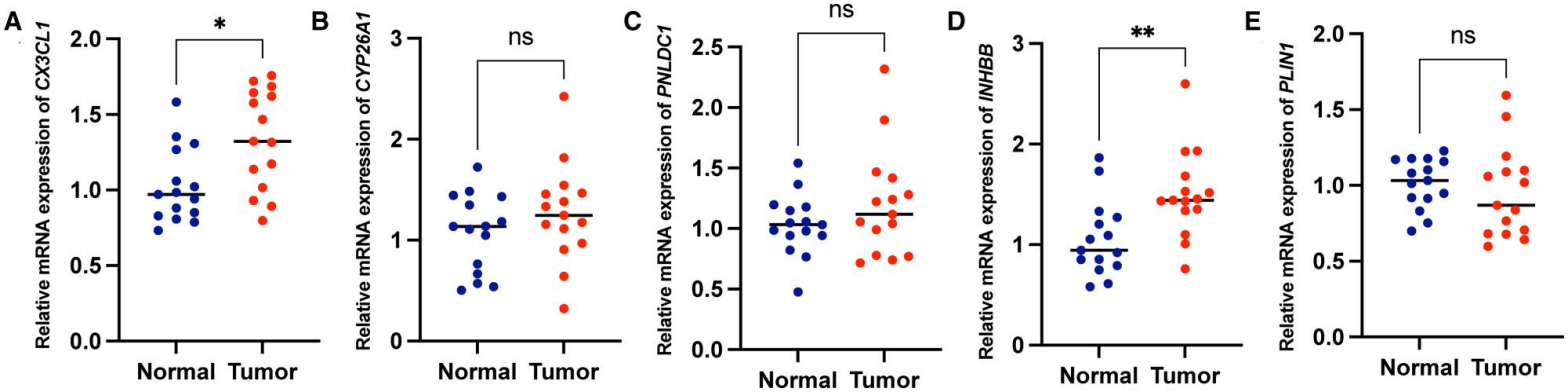
qRT–PCR. (A)–(E) Scatter plot illustrating the differential expression of prognostics genes between CRC and normal tissues. CRC = colorectal cancer, qRT–PCR = quantitative reverse transcription–PCR. *P < 0.05; **P < 0.01; ***P < 0.001.

To validate the findings derived from bioinformatics analysis, qRT–PCR analysis was conducted on CRC tissues and adjacent normal tissues collected from a cohort of 15 CRC patients. The results demonstrated upregulation of *CX3CL1* and *INHBB* expressions in tumor tissues (Figure 9).

### Analysis of enrichment and depiction of immune landscape

**Figure 10.**
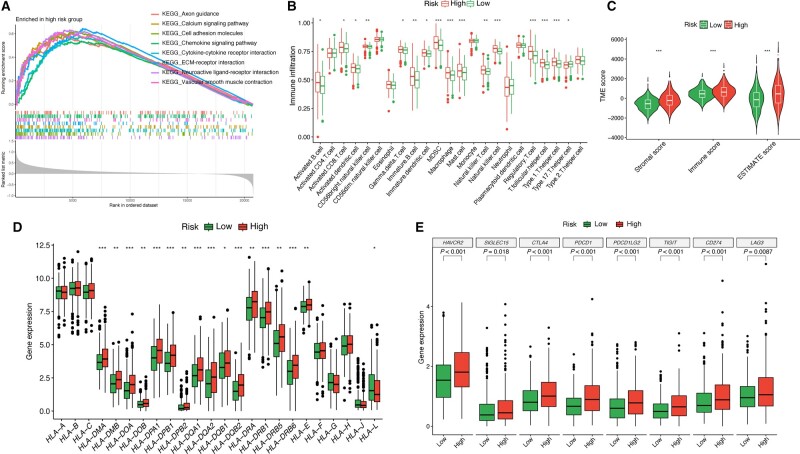
Functional and mutational analysis based on the MMRS. (A) GSEA for HRG. (B) Differences in infiltration of 23 immune cells. (C) ESTIMATE analysis. (D) Difference in major HLA-related genes between HRG and LRG. (E) Difference in major ICI-related genes between HRG and LRG. MMRS = mitophagy-mediated risk signature, HRG = high-risk group, LRG = low-risk group, ICI = immune checkpoint inhibitor, HLA = human leukocyte antigen, GSEA = gene set enrichment analysis, ESTIMATE = Estimation of STromal and Immune cells in MAlignant Tumor tissues using Expression data. *P < 0.05; **P < 0.01; ***P < 0.001.

GSEA was utilized to investigate biological functions and activated pathways for the HRG (Figure 10A). The ssGSEA and ESTIMATE algorithms were utilized to depict the immune landscape of CRC patients. A higher fraction of immune cells and ESTIMATE scores were observed in the HRG (Figure 10B and C).

### Prediction of immunotherapy response and anticancer drugs

**Figure 11.**
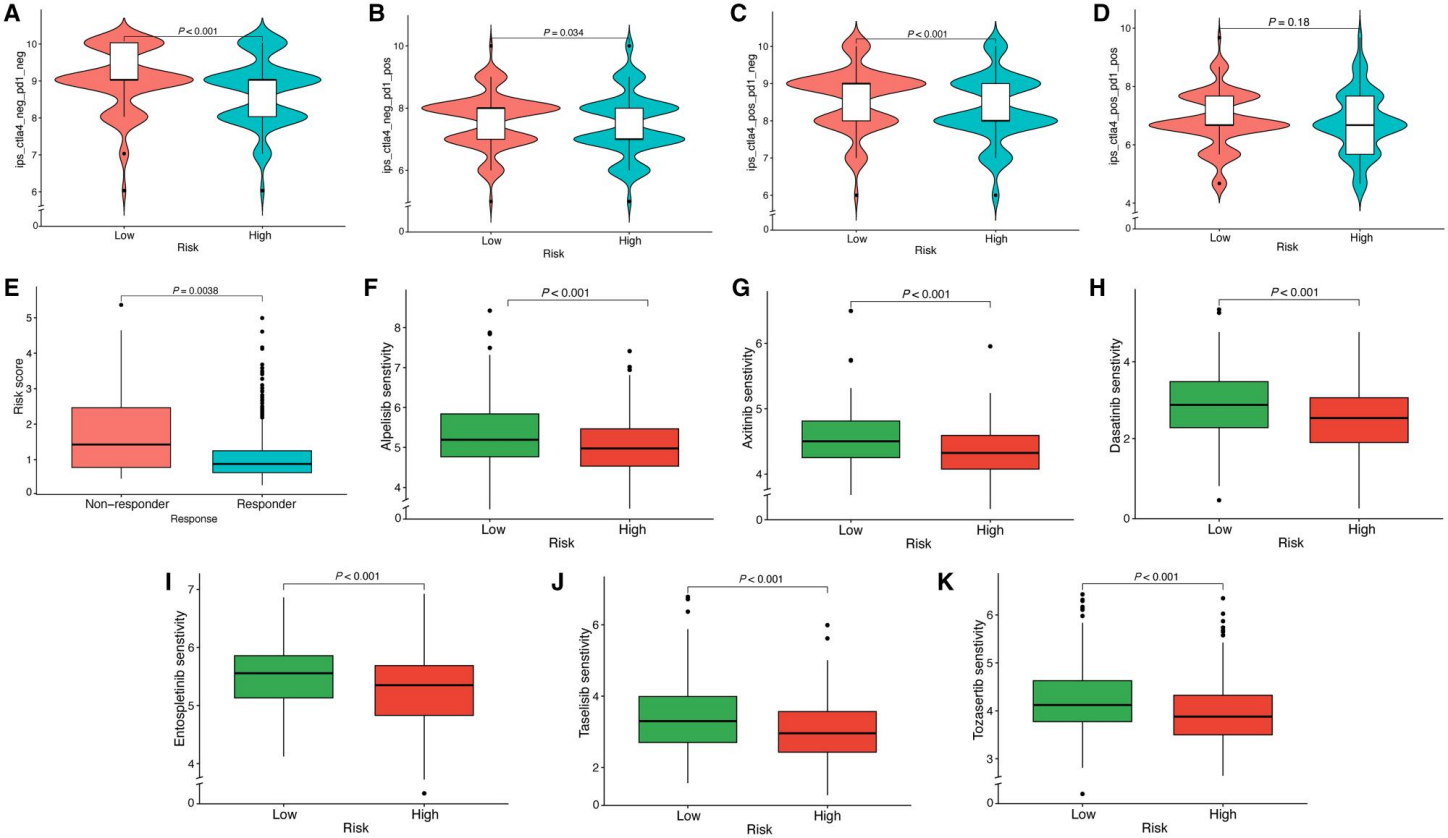
Prediction of immunotherapy response and anticancer drugs. (A)–(D) IPS score distribution map to predict immunotherapy response. (E) TIDE analysis. (F)–(K) A total of six promising anticancer drugs identified as being more sensitive in the high-risk group. IPS = immunophenoscore, TIDE = tumor immune dysfunction and exclusion.

To discover potential therapeutic targets for CRC, we examined the differential expression of ICI- and HLA-related genes between the two risk groups and observed that the HRG exhibited a higher expression level for both ICI- and HLA-related genes (Figure 10D and E). Patients in the HRG exhibited a higher immunophenoscore score compared with those in the LRG, suggesting that the LRG patients might be more responsive to ICI therapies (Figure 11A–D). Moreover, we employed TIDE analysis to predict the immunotherapy response. The lower risk score observed in the responder group suggests that the LRG patients may be more responsive to immunotherapy (Figure 11E). Furthermore, the oncoPredict algorithm predicted six anticancer drugs with lower IC50 levels in the HRG, indicating that those anticancer drugs were more likely to benefit patients in the HRG (Figure 11F–K).

## Discussion

Various factors were substantiated to contribute to the multifaceted etiology of CRC, among which mitophagy has emerged as an important contributor to tumor development and progression [24, 25]. Previous studies have pointed out that various kinds of cancers exhibit different levels of mitophagy, such as lung, breast, hepatocellular, gastric, and CRC [25–29]. However, the involved mechanisms underlying mitophagy in the TME of CRC represent an intricate process that remains largely elusive.

Using bulk RNA-seq data, a 10-gene prognostic model for predicting the OS probability of CRC was reported [30]. However, the TME is becoming an increasingly indispensable aspect of cancer research in the single-cell era. In our current work, we utilized ssGSEA to comprehensively evaluate the activity of mitophagy-related pathways at both the cellular level and the CRC tissue level. We displayed that the activity of mitophagy-related pathways was uniformly upregulated in cancer cells in the TME, while the result was verified using bulk RNA-seq data. We observed that a small subset of cancer cells exhibited downregulation of mitophagy-related pathways. Therefore, we extracted these cancer cell subpopulations and re-clustered them based on mitophagy-related genes. Upon analysing the mitophagy-related pathways in the cancer cell subpopulations, we found that the CC1 subpopulation displayed the most upregulated pathway activity, while the CC3 subpopulation showed a clear downregulation of pathway activity. Consequently, we speculate that mitophagy may play a dual role in the cancer cell environment, and this could contribute to the heterogeneity observed in CRC. To the best of our knowledge, no previous research has delved into the activity of mitophagy-related pathways within the TME or investigated the mitophagy-mediated heterogeneity of CRC.

To categorize patients for precise and optimal treatment strategies of CRC, we extracted mitophagy-related genes to divide CRC patients into two clusters: C1 and C2. We observed that C2 showed a worse prognosis and consistently higher expression of mitophagy-related genes, which led us to classify C2 as a subtype with high mitophagy activity (mitophagy-high cluster). Furthermore, the C2 cluster had a slightly higher mutation frequency compared with C1. From an immune perspective, C2 exhibited a higher immune score and increased immune cell infiltration. Moreover, the expression of ICI- and HLA-related genes was more pronounced in C2, suggesting an active immune environment and potential heightened sensitivity to immunotherapy.

To improve the clinical application of the mitophagy-related subtypes for CRC, we extracted DEGs between the two mitophagy-related clusters to develop a MMRS model. This model serves to furnish potential prognostic biomarkers for CRC that can predict the response to targeted therapy and immunotherapy treatments. The MMRS model consisted of five genes, namely *CYP26A1*, *PNLDC1*, *CX3CL1*, *INHBB*, and *PLIN1*, all of which were risk factors for CRC. CYP26A1, which is a major enzyme involved in the catabolism of retinoic acid, has been identified as a biomarker in multiple cancers. In CRC, patients with upregulated expression of *CYP26A1* have a decreased fraction of CD8+ T cells and probability of OS, indicating that targeting retinoic acid metabolism holds promise as a therapeutic approach for treating CRC [31, 32]. PNLDC1 is a poly(A)-specific ribonuclease (PARN)-like exonuclease that has been identified as being located at the mitochondrial membrane in mice [33, 34]. In CRC, it has been shown to be an RNA-binding gene associated with patient survival [35, 36]. Prior studies have provided evidence that the expression of *INHBB* and the OS probability of CRC were negatively correlated [37–39]. Intriguingly, research has confirmed the association of *INHBB* with DNA methylation changes in CRC, indicating that it is also a potential biomarker related to methylation [40]. CX3CL1 is a multifaceted chemokine that acts through a single receptor: CX3CR1 [41]. CX3CL1 has been identified as an important regulator of T-cell-mediated immunity, while its role in pro- or anti-tumorigenic immunity remains controversial across various malignancies [42–45]. Mlecnik and colleagues discovered that CX3CL1 binding to its receptor promotes the recruitment of CD8+ T cells, thereby delaying CRC recurrence [46]. We observed an upregulation of *CX3CL1* and *INHBB* expression in CRC tissues using qRT–PCR analysis of clinical samples. Therapeutic interventions targeting *CX3CL1* and *INHBB* may pave the way for innovative treatment strategies for CRC patients.

Based on the MMRS, the training cohort samples could be classified into HRG and LRG groups. We further examined the immune differences between HRG and LRG patients and found that the HRG patients demonstrated a higher immune score and more extensive immune cell infiltration. Moreover, the HRG patients exhibited a higher expression of ICI- and HLA-related genes. In conjunction with the results of the immunophenoscore and TIDE algorithms, these findings suggest that HRG patients may respond more favorably to immunotherapy. Moreover, we observed that C2 exhibited a significantly higher risk score than C1, suggesting that mitophagy might be a potential player in HRG patients. Apart from predicting immunotherapy response, we have also predicted potential anticancer drugs and identified six promising candidates, namely alpelisib, axitinib, dasatinib, entospletinib, taselisib, and tozasertib. The findings of this study hold promise for guiding personalized therapy selection for individual patients with CRC.

Admittedly, despite the promising findings, there exist several limitations to this study. First, we obtained the data from publicly available databases, which may not be fully representative of the entire patient population. Second, the results were primarily derived from bioinformatics analysis and additional validation through molecular mechanism studies is necessary. Therefore, further in-depth research is warranted to examine the underlying mechanisms of mitophagy in CRC.

## Conclusion

Collectively, we investigated the impact of mitophagy on the TME of CRC at the single-cell level. Our investigation led to the identification of two mitophagy-mediated subtypes, each exhibiting unique clinical features, biological functions, and tumor immune microenvironment features. A MMRS model consisting of five genes was developed based on the mitophagy-mediated subtypes, which exhibited excellent performance in predicting OS probability for CRC patients. Our findings may provide an innovative perspective on the complexity of the TME and identify potential therapeutic targets for CRC.

## Supplementary Material

goad066_Supplementary_Data
