# Supplementary material for: Unveiling mitophagy-mediated molecular heterogeneity and development of a risk signature model for colorectal cancer by integrated scRNA-seq and bulk RNA-seq analysis
Source: Gastroenterol Rep (Oxf). 2023 Oct 24;11:goad066. doi: 10.1093/gastro/goad066 (PMC10598840; doi:10.1093/gastro/goad066)
Supplement: goad066_Supplementary_Data [file goad066_supplementary_data.zip › Supplement.docx]

**Supplementary Table 1. Primers of qRT-PCR**

| Primer | Forward (5′ to 3′) | Reverse (5′ to 3′) |
| --- | --- | --- |
| CX3CL1 | TTCTGCCATCTGACTGTCC | TTCTGCCATCTGACTGTCC |
| CYP26A1 | GAAGAGTAAGGGTTTACTTTGC | CCCGATGTATTTAAGTTGTTCC |
| PNLDC1 | TTGATTTGCCATCGGAGTG | ACACAGACAATCCAATCTGAC |
| INHBB | GAAATCATCAGCTTCGCCG | AAGAAGTATAGGCGGACCC |
| PLIN1 | AGTTCAGTGAGGTAGCAGC | TTGGTTGAGGAGACAGCAG |
| GAPDH | TCAAGATCATCAGCAATGCC | CGATACCAAAGTTGTCATGGA |

**Supplementary Table 2. Coefficients of five prognostic genes**

| Gene | Coefficient |
| --- | --- |
| *CX3CL1* | 0.291116428 |
| *CYP26A1* | 0.689207969 |
| *PNLDC1* | 0.678813962 |
| *INHBB* | 0.245431471 |
| *PLIN1* | 0.273077755 |

**Supplementary Box. Mitophagy-related genes**

|  | | | | | |
| --- | --- | --- | --- | --- | --- |
| *BECN1* | *BOK* | *ATG7* | *OGT* | *FLCN* | *TICAM1* |
| *LRBA* | *PEX5* | *CAMKK2* | *MAP1LC3B* | *BCL2* | *CRY1* |
| *ATG4B* | *FKBP8* | *MTM1* | *SQSTM1* | *MAPT* | *FOXO1* |
| *CERS1* | *RNF5* | *ATP13A2* | *PINK1* | *SRC* | *STK11* |
| *OPTN* | *MCL1* | *SREBF2* | *ATG13ATG9A* | *AKT1* | *HGF* |
| *HUWE1* | *DCN* | *CSNK2A2* | *ATG13* | *VCP* | *MAPK3* |
| *MFN2* | *FOXO3* | *TAX1BP1* | *FIS1* | *ABL1* | *NOD2* |
| *SLC25A5* | *TSPO* | *BAG3* | *ATP5IF1* | *PSEN1* | *ATP2A2* |
| *HDAC6* | *FBXO7* | *HTT* | *ENDOG* | *ATG9A* | *SIRT2* |
| *AMBRA1* | *HAX1* | *ZFYVE1* | *SLC25A46* | *ATG9B* | *ERN1* |
| *TOMM7* | *VPS13A* | *STX17* | *PARL* | *ATG12* | *DDIT3* |
| *RIMOC1* | *BAD* | *MARK2* | *FBXL2* | *ATG5* | *PYCARD* |
| *USP30* | *CLU* | *MAP1S* | *PGAM5* | *ATG2B* | *EP300* |
| *PHB2* | *MAPK8* | *VHL* | *MUL1* | *ATG2A* | *CALM1* |
| *VDAC1* | *GBA1* | *RNF31* | *FUNDC1* | *DEPP1* | *PRKACA* |
| *ARFIP2* | *HMOX1* | *TRIM34* | *FUNDC2* | *SNX30* | *TREM2* |
| *VPS13D* | *POLDIP2* | *GABARAP* | *CISD2* | *SNX7* | *SIRT1* |
| *PRKN* | *KDR* | *CTSK* | *C19orf12* | *WIPI2* | *CDK5* |
| *VPS13C* | *VPS35* | *CAPN1* | *MOAP1* | *GABARAPL1* | *TOMM22* |
| *SPATA18* | *RNF185* | *ARSB* | *BNIP3L* | *WDR45* | *CSNK2A1* |
| *TP53* | *GSK3A* | *UCHL1* | *BNIP3* | *GABARAPL3* | *TOMM40* |
| *ATG4D* | *SESN2* | *HAP1* | *FBXL4* | *MAP1LC3B2* | *RPS27A* |
| *CDC37* | *RAB7A* | *CTTN* | *ATG3* | *WDR45B* | *UBC* |
| *RETREG1* | *LRRK2* | *FBXW7* | *CISD1* | *MAP1LC3C* | *TOMM70* |
| *SLC25A4* | *HSP90AA1* | *VPS11* | *BMF* | *WIPI1* | *MTERF3* |
| *TAFAZZIN* | *TMBIM6* | *FZD5* | *SH3GLB1* | *WDR81* | *UBB* |
| *HTRA2* | *PARK7* | *IRGM* | *HK2* | *USP36* | *MFN1* |
| *TSC2* | *MT3* | *SMURF1* | *DNM1L* | *RB1CC1* | *TOMM20* |
| *TIGAR* | *GSK3B* | *FOXK2* | *BCL2L11* | *FEZ1* | *TOMM5* |
| *SPATA33* | *SNCA* | *VMP1* | *ULK1* | *GABARAPL2* | *CSNK2B* |
| *ATG14* | *MTOR* | *SREBF1* | *NBR1* | *MAP1LC3A* | *TOMM6* |
| *RNF41* | *PRKAA1* | *HIF1A* | *STING1* | *PACS2* | *UBA52* |
