# Supplementary figures and images for: Unveiling mitophagy-mediated molecular heterogeneity and development of a risk signature model for colorectal cancer by integrated scRNA-seq and bulk RNA-seq analysis
Source: Gastroenterol Rep (Oxf). 2023 Oct 24;11:goad066. doi: 10.1093/gastro/goad066 (PMC10598840; doi:10.1093/gastro/goad066)

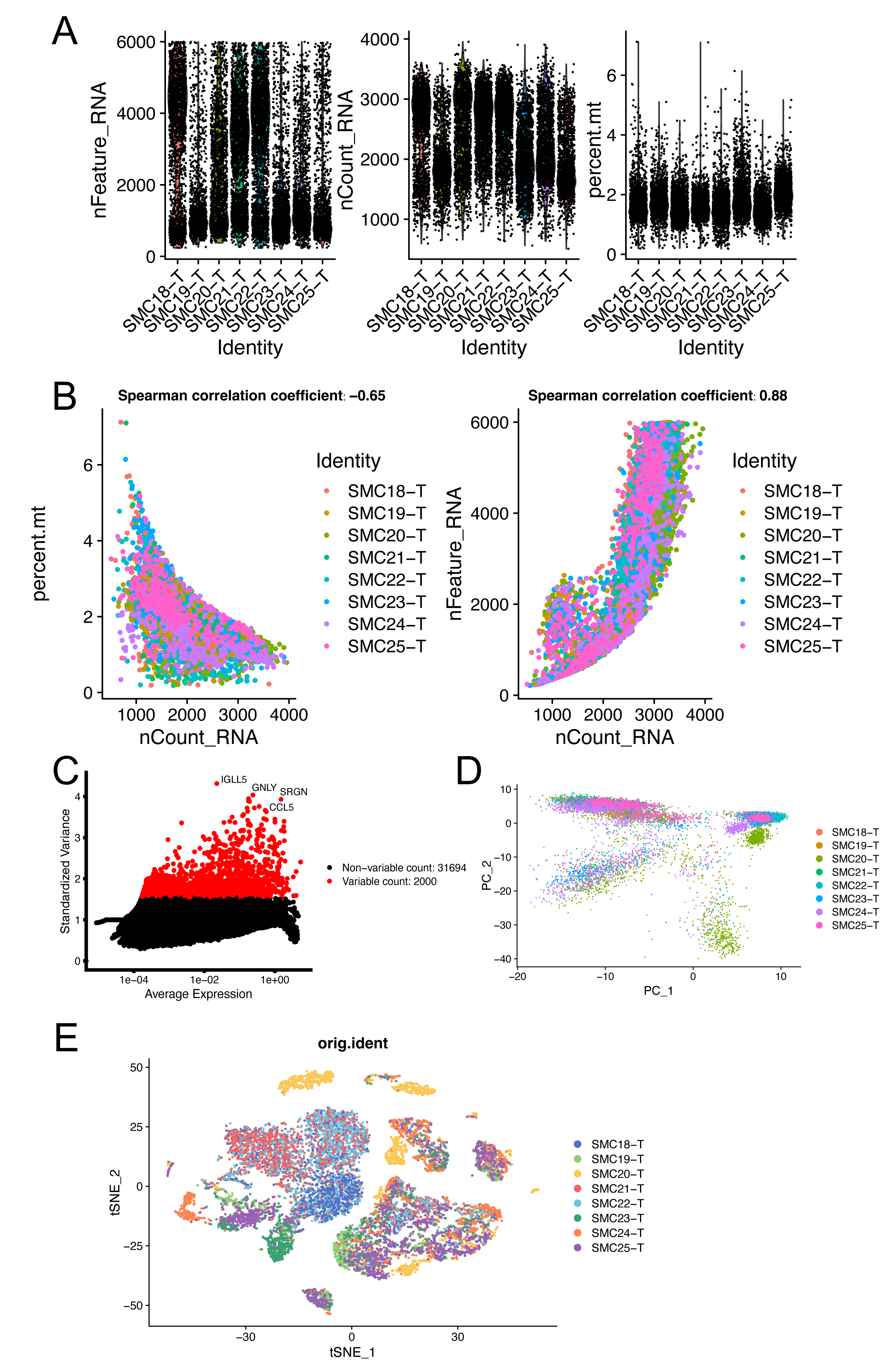

Supplement: goad066_Supplementary_Data [file goad066_supplementary_data.zip › GR-2023-201.R2_Supplementary Figure 1.tif]
